# COVID-19 vaccination in advanced skin cancer patients receiving systemic anticancer treatment: A prospective singlecenter study investigating seroconversion rates

**DOI:** 10.3389/fonc.2022.879876

**Published:** 2022-08-23

**Authors:** Georg C. Lodde, Melanie Fiedler, Ulf Dittmer, Jan-Malte Placke, Philipp Jansen, Jürgen C. Becker, Lisa Zimmer, Elisabeth Livingstone, Dirk Schadendorf, Wiebke Sondermann, Selma Ugurel

**Affiliations:** ^1^ Department of Dermatology, University Hospital Essen, Essen, Germany; ^2^ Institute for Virology, University Hospital Essen, Essen, Germany; ^3^ Translational Skin Cancer Research (TSCR), University of Duisburg/Essen, Essen, Germany; ^4^ German Consortium for Translational Cancer Research (DKTK), Partner Site Essen/Düsseldorf, Essen/Düsseldorf, Germany

**Keywords:** COVID-19 vaccination, seroconversion, skin cancer, immune checkpoint inhibition, targeted therapy

## Abstract

**Background:**

COVID-19 vaccination reduces risk of SARS-CoV-2 infection, COVID-19 severity and death. However, the rate of seroconversion after COVID-19 vaccination in cancer patients requiring systemic anticancer treatment is poorly investigated. The aim of the present study was to determine the rate of seroconversion after COVID-19 vaccination in advanced skin cancer patients under active systemic anticancer treatment.

**Methods:**

This prospective single-center study of a consecutive sample of advanced skin cancer patients was performed from May 2020 until October 2021. Inclusion criteria were systemic treatment for advanced skin cancer, known COVID-19 vaccination status, repetitive anti-SARS-CoV-2-S IgG serum quantification and first and second COVID-19 vaccination. Primary outcome was the rate of anti-SARS-CoV-2-S IgG seroconversion after complete COVID-19 vaccination.

**Results:**

Of 60 patients with advanced skin cancers, 52 patients (86.7%) received immune checkpoint inhibition (ICI), seven (11.7%) targeted agents (TT), one (1.7%) chemotherapy. Median follow-up time was 12.7 months. During study progress ten patients had died from skin cancer prior to vaccination completion, six patients were lost to follow-up and three patients had refused vaccination. 41 patients completed COVID-19 vaccination with two doses and known serological status. Of those, serum testing revealed n=3 patients (7.3%) as anti-SARS-CoV-2-S IgG positive prior to vaccination, n=32 patients (78.0%) showed a seroconversion, n=6 patients (14.6%) did not achieve a seroconversion. Patients failing serological response were immunocompromised due to concomitant hematological malignancy, previous chemotherapy or autoimmune disease requiring immunosuppressive comedications. Immunosuppressive comedication due to severe adverse events of ICI therapy did not impair seroconversion following COVID-19 vaccination. Of 41 completely vaccinated patients, 35 (85.4%) were under treatment with ICI, five (12.2%) with TT, and one (2.4%) with chemotherapy. 27 patients (65.9%) were treated non adjuvantly. Of these patients, 13 patients had achieved objective response (complete/partial response) as best tumor response (48.2%).

**Conclusion and relevance:**

Rate of anti-SARS-CoV-2-S IgG seroconversion in advanced skin cancer patients under systemic anticancer treatment after complete COVID-19 vaccination is comparable to other cancer entities. An impaired serological response was observed in patients who were immunocompromised due to concomitant diseases or previous chemotherapies. Immunosuppressive comedication due to severe adverse events of ICI did not impair the serological response to COVID-19 vaccination.

## Introduction

Patients with active cancer disease were reported to be at risk of poor outcomes from severe acute respiratory syndrome coronavirus type 2 (SARS-CoV-2) infection with an increased rate of severe courses and deaths from coronavirus disease 2019 (COVID-19) (1–3). Clinical trials of different COVID-19 vaccines (mRNA-1273, Moderna, Cambridge, USA; BNT162b24, BioNTech-Pfizer, Mainz, Germany; AZD1222, AstraZeneca, Oxford, UK) have demonstrated a reduced risk of SARS-CoV-2 infection and COVID-19 severity and death (4–6). However, these trials did not report on vaccination outcomes of cancer patients. A recent single-center study from Boston, USA reported impaired anti-SARS-CoV-2 antibody responses towards different COVID-19 vaccines in patients with various types of solid and hematologic cancers, describing an association of inferior serological response with the use of chemotherapies and corticosteroids (7). The aim of the present study was to determine the rate of seroconversion after COVID-19 vaccination in advanced skin cancer patients under active systemic anticancer treatment.

## Materials and methods

### Study design and patient eligibility

**Figure 1:**
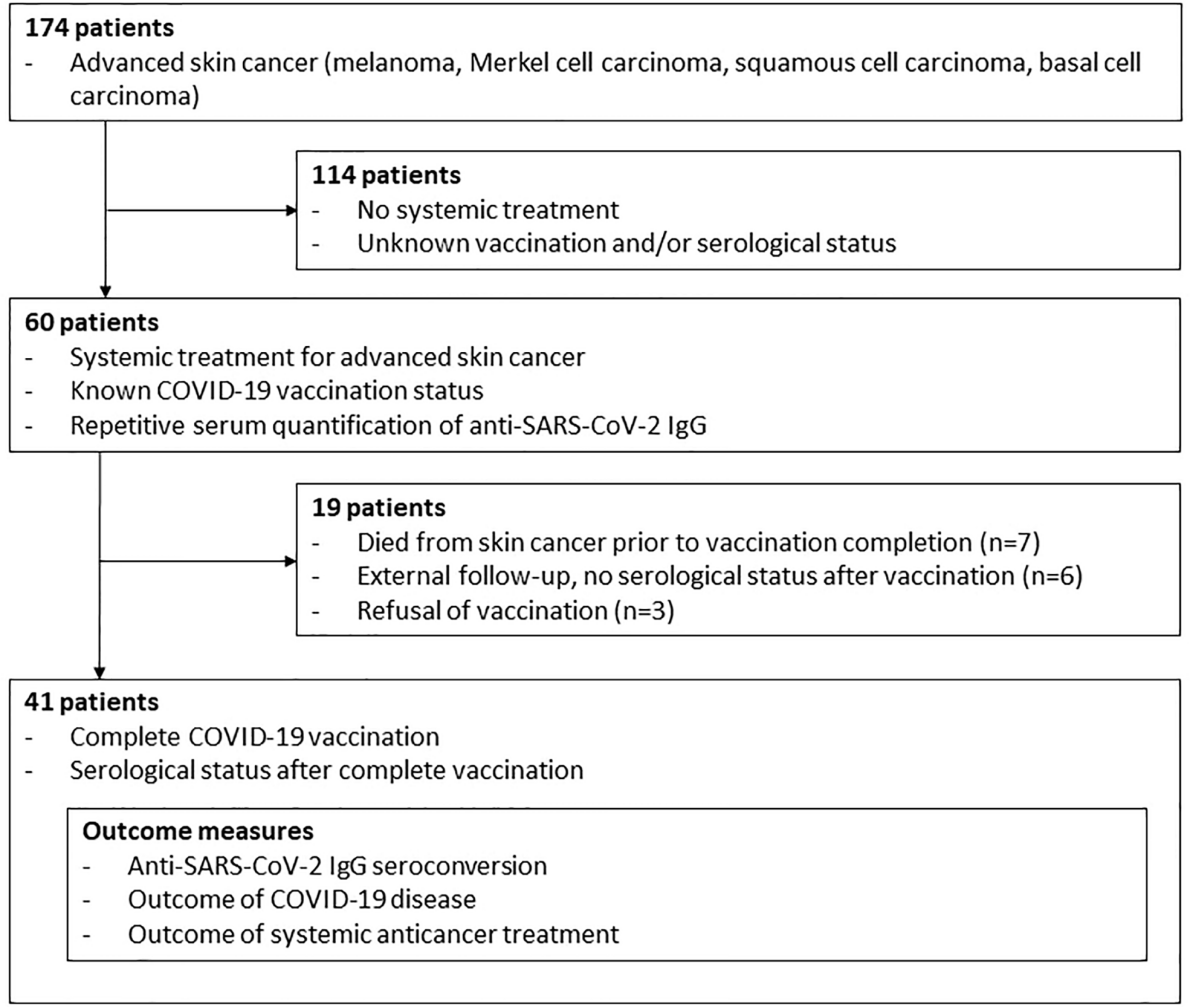
Patient Flow.

This prospective single-center study of a consecutive sample of advanced skin cancer patients was performed from May 2020 until October 2021 at the Department of Dermatology, University Hospital Essen. Inclusion criteria were systemic treatment for advanced skin cancer, known COVID-19 vaccination status, repetitive anti-SARS-CoV-2-S IgG serum quantification and first and second COVID-19 vaccination. Advanced skin cancer patients without systemic treatment, unknown COVID-19 vaccination status and incomplete COVID-19 vaccination were excluded from the study (**Figure 1**). Primary outcome was the rate of seroconversion in advanced skin cancer patients with systemic anticancer treatment after second dose of COVID-19 vaccination. Data on patient and tumor characteristics, concomitant diseases, immunosuppressive comedications, systemic anticancer therapy, and COVID-19 vaccination status were collected. Systemic anticancer therapies included immune checkpoint inhibition (ICI; anti-PD-1/anti-PD-L1/anti-CTLA-4 antibodies), targeted therapy (TT; BRAF+MEK kinase inhibition), and chemotherapy. Concomitant diseases were evaluated using the modified Charlson comorbidity index (CCI) (8). Primary study outcome was the rate of anti-SARS-CoV-2-S IgG seroconversion after second COVID-19 vaccination. Anti-SARS-CoV-2-S antibodies were measured at each dose (ICI) or cycle (TT, chemotherapy) of ongoing anticancer therapy, which corresponds to an at least monthly testing. In cases of treatment completion, serum testing was performed at follow-up visits in 3-months intervals. Secondary study outcome was best tumor response achieved to systemic anticancer treatment within the observation time of the study. It was measured by the institutional interdisciplinary tumor board as physician’s assessment according to RECIST (9) within regular clinical practice in staging intervals.

### Serum antibody testing

IgG antibodies against SARS-CoV-2 spike protein (anti-SARS-CoV-2-S IgG) in patients’ sera were measured by chemiluminescence assays (SARS-CoV-2 S1/S2 IgG or SARS-CoV-2 TrimericS IgG, DiaSorin, Saluggia, Italy) using the LIASION-XL (DiaSorin) following the manufacturer's instructions. The first assay is only semi-quantitative, whereas the second one is quantitative and adjusted to the upcoming WHO standard. Values >15 AU/ml corresponding to 39 BAU/ml, and values >33.8 BAU/ml were considered positive, respectively. Sensitivity/specificity for each assay are 94,4%/98,6% and 96,9%/100%, respectively. For this study only qualitative results were used to document seroconversion.

### Data analysis

The study was approved by the ethics committee of the University Duisburg-Essen (21-10141-BO). The period between first serum testing and last patient visit was considered as follow-up time. Descriptive statistics were performed using SPSSv26.0.

## Results

### Total study cohort

At start of this study, 60 patients with advanced skin cancers received systemic anticancer treatment (**Figure 1**). Median follow-up time was 12.7 months. Patients were treated for melanoma (n=51, 85.0%), Merkel cell carcinoma (n=6, 10.0%), cutaneous squamous cell carcinoma (n=2, 3.3%), and basal cell carcinoma (n=1, 1.7%); **Table 1**. Most patients had a modified CCI of 0 (n=44, 73.3%), and an unimpaired performance status (n=53, 88.3%). 52 patients (86.7%) received ICI therapy, seven patients (11.7%) received TT, and one patient received chemotherapy. Systemic anticancer treatment was given to 41 patients (68.3%) in a non-adjuvant setting.

**Table 1:** Patient characteristics.

	Total study cohort N (%)	COVID-19 vaccinated N (%)
Total	60 (100.0)	41 (100.0)
Median age, years (range)	65.0 (41–80)	64.0 (41–80)
Sex		
Female	25 (41.7)	17 (51.5)
Male	35 (58.3)	24 (58.5)
Type of skin cancer		
Melanoma	51 (85.0)	37 (90.2)
Merkel cell carcinoma	6 (10.0)	2 (4.9)
Squamous cell carcinoma	2 (3.3)	1 (2.4)
Basal cell carcinoma	1 (1.7)	1 (2.4)
Charlson comorbidity index^1^		
0	44 (73.3)	31 (75.6)
1-2	13 (21.7)	9 (22.0)
≥3	3 (5.0)	1 (2.4)
Overall performance status (ECOG)		
0	53 (88.3)	38 (92.7)
1-2	6 (10.0)	1 (2.4)
≥3	1 (1.7)	2 (4.9)
LDH (serum)		
Normal	48 (80.0)	35 (85.4)
Increased	12 (20.0)	6 (14.6)
Number of organs involved		
0	19 (31.7)	14 (34.1)
1-3	32 (53.3)	23 (56.1)
>3	9 (15.0)	4 (9.8)
Type of systemic treatment		
Immune checkpoint inhibition	52 (86.7)	35 (85.4)
Monotherapy (PD-1, PD-L1)	38 (63.3)	26 (63.4)
Combination (CTLA-4+PD-1)	14 (23.3)	9 (22.0)
Targeted therapy (BRAF+MEK)	7 (11.7)	5 (12.2)
Chemotherapy	1 (1.7)	1 (2.4)
Treatment setting		
Adjuvant	19 (31.7)	14 (34.1)
Non-adjuvant	41 (68.3)	27 (65.9)
Treatment line		
First-line	35 (58.3)	27 (65.9)
Second-line or higher	25 (41.7)	14 (34.1)
Tumor response to systemic treatment		
Complete response	2 (3.3)	2 (3.3)
Partial response	15 (25.0)	11 (26.8)
Stable disease	6 (10.0)	4 (9.8)
Progressive disease	17 (28.3)	9 (22.0)
No evidence of disease	17 (28.3)	12 (29.3)
Not evaluable	3 (5.0)	3 (7.3)
Survival status		
Alive	50 (83.3)	40 (97.6)
Dead	10 (16.7)	1 (2.4)
Died from skin cancer	10 (16.7)	1 (2.4)
Died from COVID-19	0 (0.0)	0 (0.0)

Characteristics of 60 patients receiving systemic treatment for advanced skin cancer at the time of first serum testing for anti-SARS-CoV-2-S antibodies. The outcome of systemic treatment and the patients’ survival status during study progress are also provided. ^1^ modified, the underlying skin cancer was excluded from comorbidities. LDH, lactate dehydrogenase.

At database closure on October 15, 2021, one patient had developed a symptomatic COVID-19 infection. At the time of symptomatic infection, this patient received a systemic treatment with CTLA-4+PD-1 for advanced melanoma. The laboratory data showed normal values for neutrophil granulocytes (5.27/nl, reference 1.7-6.2/nl), lymphocytes (1.42/nl, reference 1.0-3.4/nl) and LDH (234 U/l, reference 120-247 U/l). The patient suffered from mild clinical symptoms including taste disorders, glossodynia and dry coughing. Symptoms were declining spontaneously and the patient was vaccinated four months later when vaccines were available. None of the patients had died from COVID-19.

### Patients with serological status after complete COVID-19 vaccination

During study progress ten patients had died from skin cancer prior to vaccination completion, six patients were lost to follow-up and three patients had refused vaccination due to fear of negative interaction with their anticancer treatment or of severe side effects (**Figure 1**). 41 patients completed COVID-19 vaccination, corresponding to two sequential mRNA and viral vector vaccine applications or a combination of viral vector and mRNA vaccine. From these 41 patients, serological status was known after complete vaccination. 35 (85.4%) were under treatment with ICI, five (12.2%) with TT, and one (2.4%) with chemotherapy (**Table 1**).

After complete COVID-19 vaccination, 32 patients (78.0%) showed an anti–SARS-CoV-2-S IgG seroconversion (**Table 2**). Seroconversion was achieved in 26/35 patients treated with ICI, 5/5 patients treated with TT, and 1/1 patient treated with chemotherapy. In three patients (7.3%) anti-SARS-CoV-2-S IgG was detected prior to vaccination, indicating previous SARS-CoV-2 infections which were asymptomatic and previously unknown to the respective patients. At the time of first COVID-19 vaccination, nine patients had active immunosuppressive comedications (**Table 2**). None of these nine patient were neutropenic at the time of vaccination (reference >1.7/nl). Seven patients received immunosuppressive comedications for treatment of severe adverse events of ICI (corticosteroids, n=6; extracorporeal photopheresis, n=1). All of these seven patients showed anti-SARS-CoV-2-S IgG seroconversion after complete vaccination.

**Table 2:** COVID-19 vaccination and outcome.

	N
Total	41
Vaccination type (first and second vaccination)	
mRNA (2x mRNA-1273, n=1; 2x BNT162b24, n=36)	37
Viral vector (2x AZD1222)	2
Mixed (1x viral vector, 1x mRNA)	2
Immunosuppressive comedication	
None	32
Corticosteroids (methylprednisolon ≥1mg/kg/d, prednisolon ≥100mg/d)	6
Rituximab	1
Extracorporeal photopheresis	1
Ustekinumab	1
Anti-SARS-CoV-2-S IgG (serum)	
Positive prior to vaccination	3
Positive after vaccination (seroconversion)	32
Immune checkpoint inhibition	26
Targeted therapy (BRAF+MEK)	5
Chemotherapy	1
Not positive after vaccination (no seroconversion)	6
Immune checkpoint inhibition	6
MM; concomitant chronic inflammatory bowel disease (M. Crohn); active immunosuppressive therapy (IL-12-/IL-23 monoclonal antibody)	1
MM; concomitant malignancy (mantle cell lymphoma); previous chemotherapy (R-CHOP, R-DHAP) and autologous stem cell transplantation; active immunosuppressive therapy (monoclonal CD-20 antibody)	1
MM; previous chemotherapy (dacarbazine), last treatment 12 months prior to time of vaccination	1
MM; concomitant malignancy (colorectal carcinoma); previous chemotherapy (FOLFOX), last treatment 33 months prior to time of vaccination	1
cSCC; concomitant malignancy (chronic lymphocytic leukemia); previous chemotherapy (cyclophosphamide, methotrexate, fluorouracil), last treatment 15 years prior to time of vaccination	1
MM; concomitant malignancy (chronic lymphocytic leukemia)	1

Characteristics of 41 advanced skin cancer patients with active anticancer therapy who completed COVID-19 vaccination during study progress, corresponding to the time of first vaccine application. Concomitant diseases and comedications of patients failing seroconversion are shown in detail. Abbreviations: MM, melanoma; cSCC, cutaneous squamous cell carcinoma; R-CHOP, rituximab, cyclophosphamide, doxorubicin, vincristine, prednisolone; R-DHAP, rituximab, dexamethasone, cytarabine, cisplatin; FOLFOX, folinic acid, fluorouracil, oxaliplatin.

In six of 41 completely vaccinated patients (14.6%) anti-SARS-CoV-2-S IgG antibodies could not be detected in repeated serological testings after vaccination (**Table 2**; **Supplement Table 1**). These patients failing seroconversion received ICI (CTLA-4+PD-1, n=2; PD-1, n=4) in time of vaccination. Two patients were treated with immunosuppressive comedications due to concomitant diseases with timing of vaccination.

One patient received IL-12 and IL-23 monoclonal antibody for treatment of chronic inflammatory bowel disease (M. Crohn) for more than 16 months without neutropenia (reference >1.7/nl). Furthermore, the patient had a known Turner syndrome.

The second patient received chimeric monoclonal CD-20 antibody for treatment of mantle cell lymphoma at the time of COVID-19 vaccination. The patient’s history revealed first diagnosis of mantle cell lymphoma, stage IV Ann Arbor disease (10), 20 months prior to first COVID-19 vaccination. At time of first diagnosis of mantle cell lymphoma this patient had been treated with a R-CHOP chemotherapy for five months. Autologous stem cell transplantation was performed 15 months prior to COVID-19 vaccination with subsequently CD-20 antibody treatment. This patient had a lymphopenia (0.37/nl, reference 1.0-3.4/nl) with normal values of neutrophil granulocytes (4.14/nl, reference 1.7-6.2/nl) in time of vaccination.

The third patient without seroconversion had a palliative melanoma disease and multiple systemic treatment lines including darcabazine chemotherapy 12 months before COVID-19 vaccination. This patient had a lymphopenia (0.70nl, reference 1.0-3.4/nl) with slightly increased neutrophil granulocytes (6.43/nl, reference 1.7-6.2/nl).

The fourth patient without seroconversion was diagnosed with rectum carcinoma 38 months prior to first COVID-19 vaccination. After total excision of rectum carcinoma the patient had received FOLFOX chemotherapy as adjuvant treatment for five months. In time of vaccination differential blood showed no neutropenia nor lymphopenia.

The fifth patient with failed seroconversion was diagnosed with chronic lymphocytic leukemia 16 years prior to first vaccination. This patient had been treated with chemotherapy (cyclophosphamide, methotrexate, fluorouracil) after first diagnosis of chronic lymphocytic leukemia for six months. At time of vaccination the patient had normal values for neutrophile granulocytes and lymphocytes.

The last patient without seroconversion had a chronic lymphocytic leukemia, Binet stage A (11). This patient presented with leukocytosis (26.7/nl, reference 3.6-9.2) and lymphocytosis (13.9/nl; reference 1-4/nl) at time of vaccination.

Of 41 completely vaccinated patients, 27 patients (65.9%) were treated non adjuvantly. Of these patients, 13 patients had achieved an objective response as best tumor response (48.2%).

Of the 27 patients treated non adjuvantly, 21 patients had a melanoma. Objective response as best tumor response in exclusively non adjuvantly treated melanoma patients was 47.6% (n=10).

## Discussion

In our consecutive cohort, 78.0% of skin cancer patients achieved an anti–SARS-CoV-2-S IgG seroconversion after complete COVID-19 vaccination. Similar response rates have been described previously for patients with different cancer entities without immunosuppressive treatment (8, 12). However, as skin cancers are solid tumors, the rate of seroconversion was lower compared to the study of Thakkar et al. (13).

Only one patient developed symptomatic COVID-19, and no patient died from COVID-19 during study course.

With regard to anticancer treatment, the treatment outcomes observed in our investigated patient cohort are comparable to those reported from similar cohorts before the COVID-19 pandemic. Moreover, the anticancer treatment outcomes of completely vaccinated patients were not inferior to those of incompletely or not vaccinated patients. For non-adjuvant melanoma patients treated with ICI (n=21) we observed objective response rates comparable to previously reported responses in metastatic melanomas (14). Thus, our results strengthen the recent suggestion that advanced skin cancer patients should be offered treatment with ICI or TT in times of the pandemic without delay, as there is currently no evidence that this would increase the risk of severe COVID-19 (15, 16). However, our study is still limited by the low number of patients and the heterogeneity of baseline characteristics.

14.6% of the investigated patients did not develop an anti-SARS-CoV-2-S IgG antibody response despite complete vaccination. All of these patients were under active ICI treatment, yet their failure of seroconversion was likely caused by their underlying immunocompromised status as these patients either had a concomitant hematological malignancy, a previously obtained chemotherapy, or an immunosuppressive comedication for concomitant autoimmune disease. This observation is in line with recently published data showing lower COVID-19 vaccination efficacy in immunocompromised patients (17). One study reported of seropositive response rates of 76% for patients with active myeloma compared to seropositive response rates of 98% in a healthy cohort study (12). A recently published study described lower rates of seroconversion in patients who underwent immunosuppressive treatments as stem cell transplant or CD-20-antibody treatment (13, 18). Two of six patients with impaired serological response had active immunosuppressive treatment (IL-12-/IL-23 monoclonal antibody, monoclonal CD-20 antibody) in time of vaccination (**Table 1**). Time since last administration of immunosuppressive therapy might be associated with seroconversion after vaccination (18). However, number of patients is too low for further analyzes of time of vaccination and time of administered immunosuppressive treatment.

In two of six patients with impaired serological response we detected a lymphopenia (<0.37/nl, reference 1.0-3.4/nl). One patient was treated with monoclonal CD-20 antibody for mantle cell lymphoma (mentioned above). The other patient was in palliative metastatic melanoma setting. Lymphopenia has been described previously as risk factor for impaired serological response in hematological malignancies (12). 17.1% (7/41) of the completely vaccinated patients of our study cohort required immunosuppressive comedication, mostly corticosteroids, for the treatment of severe adverse events of ICI therapy. Notably, none of these patients failed to develop an anti-SARS-CoV-2-S IgG seroconversion after COVID-19 vaccination. Management of adverse events of ICI therapy with corticosteroids may not reduce the rate of seroconversion and presents no contraindication for vaccination against COVID-19 (13). This finding begs the question if a delay in COVID-19 vaccination for patients under active treatment with corticosteroids for ICI adverse events is still recommendable (17).

The majority of patients received ICI therapy. ICI therapy may induce autoimmune side effects as pneumonitis (19, 20). Clinical symptoms of autoimmune pneumonitis overlaps with COVID-19 pneumonia. The indistinguishable clinical symptoms of autoimmune induced pneumonitis and COVID-19 pneumonia could lead to undetected breakthrough infections of COVID-19 (21).

Limitations of our study include the relatively small number of patients and the monocentric design. A direct comparison between subgroups of the study cohort is not valid due to the heterogeneity of baseline characteristics with respect to type of skin cancer, tumor stage, treatment setting and type of systemic treatment. Other limitations include the lack of a control group and of cellular data as B cell numbers and T cell response. The field of SARS-CoV-2 diagnostic is highly dynamic. Therefore, we had to use two test generations in this prospective longitudinal study. The first assay is only semi-quantitative, whereas the second one is quantitative and adjusted to the upcoming WHO standard. Serological response to COVID-19 vaccinations in advanced skin cancer patients was analyzed. This study is limited to analyze the efficacy of COVID-19 vaccination in skin cancer patients. Seroconversion has not been correlated with clinical outcomes as hospitalization and mortality rate. However, serological response might be associated with clinical outcomes and could be used to estimate the efficacy of COVID-19 vaccination (22, 23). Larger real-world studies are needed to confirm our preliminary findings.

## Conclusion

Rate of seroconversion to COVID-19 vaccination is high in advanced skin cancer patients receiving active systemic anticancer treatment. An impaired serological response to COVID-19 vaccination was observed in patients who were immunocompromised due to concomitant hematological malignancies, previous chemotherapies, or immunosuppressive comedication for underlying autoimmune disease. Lymphopenia might be a risk factor for an impaired serological response to COVID-19 vaccination. Immunosuppressive comedication, in particular by corticosteroids, for treatment of severe side effects of anticancer immunotherapy, did not impair the serological response to COVID-19 vaccination.

## Data availability statement

The raw data supporting the conclusions of this article will be made available by the authors, without undue reservation.

## Ethics statement

The study was approved by the institutional ethics committee of the University Duisburg-Essen (21-10141-BO). It was conducted in accordance with the Declaration of Helsinki. Written informed consent for participation was not required for this study in accordance with the national legislation and the institutional requirements.

## Author contributions

Conceptualization: SU, WS, and GL.; methodology: SU, MF, WS, and GL; formal analysis: MF, SU, WS, and GL; resources: UD, LZ, DS, EL, WS, SU, and GL; data curation, GL and SU.; writing - original draft preparation: GL and SU; writing and editing: GL, MF, UD, J-MP, PJ, JB, LZ, DS, EL, WS, and SU; visualization: SU, GL, and WS.; supervision: SU; project administration: GL, SU, and WS; all authors have read and agreed to the final version of the manuscript.

## Acknowledgments

We thank all study participants and their families. Part of this work was funded by the Deutsche Forschungsgemeinschaft (DFG, German 582 Research Foundation, RTG 2535, Knowledge- and data-driven personalization of medicine at the 583 point of care). J-MP was supported by the DFG in the framework of the DFG Clinician Scientist Programme UMEA, FU 356/12-1).

## Conflict of interest

GL has received travel support from Sun Pharma. MF has given a paid lecture for Dia Sorin. J-MP served as consultant and/or has received honoraria from Bristol-Myers Squibb and Novartis, and received travel support from Bristol-Myers Squibb, Novartis and Therakos. JB declares speaker honoraria from Amgen, MerckSerono, Pfizer, Sanofi; advisory board honoraria from 4SC, Amgen, CureVac, eTheRNA, MerckSerono, Novartis and InProTher; research funding from Alcedis, Boehringer Ingelheim, Bristol-Myers Squibb, IQVIA, and MerckSerono; travel support from 4SC and Incyte. LZ served as consultant and/or has received honoraria from Bristol-Myers Squibb, Merck Sharp & Dohme, Novartis, Pierre-Fabre, Sunpharma and Sanofi; Research funding to institution: Novartis; travel support from Merck Sharp & Dohme, Bristol-Myers Squibb, Amgen, Pierre-Fabre, Sanofi, Sunpharma and Novartis, outside the submitted work. EL served as consultant and/or has received honoraria from Bristol-Myers Squibb, Merck Sharp & Dohme, Novartis, Medac, Pierre Fabre, Sanofi, Sunpharma and travel support from Amgen, Merck Sharp & Dohme, Bristol-Myers Squibb, Pierre Fabre, Sunpharma and Novartis, outside the submitted work. DS declares relevant financial activities with Roche, Novartis, Bristol-Myers Squibb, Merck Sharp & Dohme, Sanofi, Regeneron, Pfizer, Array, Pierre Fabre, 4SC, Helsinn, Philogen, InFlarX, Merck-Serono, SunPharma, Ultimovacs, and Sandoz. WS reports grants from medi GmbH Bayreuth, grants and personal fees from Novartis and Almirall, personal fees from Abbvie, Amgen, GSK, Lilly, UCB, LEO Pharma, Sanofi, Genzyme, and Janssen outside the submitted work. SU declares research support from Bristol Myers Squibb and Merck Serono; speakers and advisory board honoraria from Bristol Myers Squibb, Merck Sharp & Dohme, Merck Serono, Novartis and Roche, and travel support from Bristol Myers Squibb, Merck Sharp & Dohme, and Pierre Fabre; outside the submitted work.

The remaining authors declare that the research was conducted in the absence of any commercial or financial relationships that could be construed as a potential conflict of interest.

## Publisher’s note

All claims expressed in this article are solely those of the authors and do not necessarily represent those of their affiliated organizations, or those of the publisher, the editors and the reviewers. Any product that may be evaluated in this article, or claim that may be made by its manufacturer, is not guaranteed or endorsed by the publisher.

## References

[1] Kuderer NM Choueiri TK Shah DP Shyr Y Rubinstein SM Rivera DR. Clinical impact of covid-19 on patients with cancer (Ccc19): A cohort study. Lancet (2020) 395(10241):1907–18. doi: 10.1016/S0140-6736(20)31187-9 PMC7255743 32473681

[2] Desai A Sachdeva S Parekh T Desai R. Covid-19 and cancer: Lessons from a pooled meta-analysis. JCO Global Oncol (2020) 6):557–9. doi: 10.1200/go.20.00097 PMC7193801 32250659

[3] Richardson S Hirsch JS Narasimhan M Crawford JM McGinn T Davidson KW. Presenting characteristics, comorbidities, and outcomes among 5700 patients hospitalized with covid-19 in the New York City area. JAMA (2020) 323(20):2052–9. doi: 10.1001/jama.2020.6775 PMC7177629 32320003

[4] Baden LR El Sahly HM Essink B Kotloff K Frey S Novak R. Efficacy and safety of the mrna-1273 sars-Cov-2 vaccine. N Engl J Med (2020) 384(5):403–16. doi: 10.1056/NEJMoa2035389 PMC7787219 33378609

[5] Polack FP Thomas SJ Kitchin N Absalon J Gurtman A Lockhart S. Safety and efficacy of the Bnt162b2 mrna covid-19 vaccine. N Engl J Med (2020) 383(27):2603–15. doi: 10.1056/NEJMoa2034577 PMC7745181 33301246

[6] Voysey M Clemens SAC Madhi SA Weckx LY Folegatti PM Aley PK. Safety and efficacy of the Chadox1 ncov-19 vaccine (Azd1222) against sars-Cov-2: An interim analysis of four randomised controlled trials in Brazil, South Africa, and the UK. Lancet (2021) 397(10269):99–111. doi: 10.1016/S0140-6736(20)32661-1 33306989 PMC7723445

[7] Naranbhai V Pernat CA Gavralidis A St Denis KJ Lam EC Spring LM. Immunogenicity and reactogenicity of sars-Cov-2 vaccines in patients with cancer: The canvax cohort study. J Clin Oncol (2022) 40(1):12–23. doi: 10.1200/jco.21.01891 34752147 PMC8683230

[8] Thomas SJ. 1558o - covid-19 vaccine in participants (Ptcpts) with cancer: Subgroup analysis of Efficacy/Safety from a global phase III randomized trial of the Bnt162b2 (Tozinameran) mrna vaccine. Ann Oncol (2021) 32(suppl_5):S1129-S1163 101016/annonc/annonc713. doi: 10.1016/j.annonc.2021.08.1551

[9] Schwartz LH Litière S de Vries E Ford R Gwyther S Mandrekar S. Recist 1.1-update and clarification: From the recist committee. Eur J Cancer (2016) 62:132–7. doi: 10.1016/j.ejca.2016.03.081 PMC5737828 27189322

[10] Armitage JO. Staging non-Hodgkin lymphoma. CA: A Cancer J Clin (2005) 55(6):368–76. doi: 10.3322/canjclin.55.6.368 16282281

[11] Binet JL Auquier A Dighiero G Chastang C Piguet H Goasguen J. A new prognostic classification of chronic lymphocytic leukemia derived from a multivariate survival analysis. Cancer (1981) 48(1):198–206. doi: 10.1002/1097-0142(19810701)48:1<198::aid-cncr2820480131>3.0.co;2-v 7237385

[12] Avivi I Balaban R Shragai T Sheffer G Morales M Aharon A. Humoral response rate and predictors of response to Bnt162b2 mrna Covid19 vaccine in patients with multiple myeloma. Br J Haematology (2021) 195(2):186–93. doi: 10.1111/bjh.17608 PMC8444771 34196388

[13] Thakkar A Gonzalez-Lugo JD Goradia N Gali R Shapiro LC Pradhan K. Seroconversion rates following covid-19 vaccination among patients with cancer. Cancer Cell (2021) 39(8):1081–90.e2. doi: 10.1016/j.ccell.2021.06.002 34133951 PMC8179248

[14] Larkin J Chiarion-Sileni V Gonzalez R Grob J-J Rutkowski P Lao CD. Five-year survival with combined nivolumab and ipilimumab in advanced melanoma. N Engl J Med (2019) 381(16):1535–46. doi: 10.1056/NEJMoa1910836 31562797

[15] Switzer B Haanen J Lorigan PC Puzanov I Turajlic S. Clinical and immunologic implications of covid-19 in patients with melanoma and renal cell carcinoma receiving immune checkpoint inhibitors. J ImmunoTherapy Cancer (2021) 9(7):e002835. doi: 10.1136/jitc-2021-002835 PMC8288220 34272309

[16] Gambichler T Reuther J Scheel CH Becker JC. On the use of immune checkpoint inhibitors in patients with viral infections including covid-19. J ImmunoTherapy Cancer (2020) 8(2):e001145. doi: 10.1136/jitc-2020-001145 PMC7358098 32611687

[17] Romano E Pascolo S Ott P. Implications of mrna-based sars-Cov-2 vaccination for cancer patients. J ImmunoTherapy Cancer (2021) 9(6):e002932. doi: 10.1136/jitc-2021-002932 PMC8206178 34117117

[18] Ollila TA Lu S Masel R Zayac A Paiva K Rogers RD. Antibody response to covid-19 vaccination in adults with hematologic malignant disease. JAMA Oncol (2021) 7(11):1714–6. doi: 10.1001/jamaoncol.2021.4381 PMC8358793 34379085

[19] Heinzerling L de Toni E Schett G Hundorfean G Zimmer L. Checkpoint inhibitors. Dtsch Arztebl Int (2019) 116(8):119–26. doi: 10.3238/arztebl.2019.0119 PMC6454802 30940340

[20] Eigentler TK Hassel JC Berking C Aberle J Bachmann O Grünwald V. Diagnosis, monitoring and management of immune-related adverse drug reactions of anti-Pd-1 antibody therapy. Cancer Treat Rev (2016) 45:7–18. doi: 10.1016/j.ctrv.2016.02.003 26922661

[21] Abid MB. Overlap of immunotherapy-related pneumonitis and covid-19 pneumonia: Diagnostic and vaccine considerations. J Immunother Cancer (2021) 9(4):e002307. doi: 10.1136/jitc-2020-002307 33931473 PMC8098953

[22] Khoury DS Cromer D Reynaldi A Schlub TE Wheatley AK Juno JA. Neutralizing antibody levels are highly predictive of immune protection from symptomatic sars-Cov-2 infection. Nat Med (2021) 27(7):1205–11. doi: 10.1038/s41591-021-01377-8 34002089

[23] Earle KA Ambrosino DM Fiore-Gartland A Goldblatt D Gilbert PB Siber GR. Evidence for antibody as a protective correlate for covid-19 vaccines. Vaccine (2021) 39(32):4423–8. doi: 10.1016/j.vaccine.2021.05.063 PMC8142841 34210573

