# Supplementary material for: COVID-19 vaccination in advanced skin cancer patients receiving systemic anticancer treatment: A prospective singlecenter study investigating seroconversion rates
Source: Front Oncol. 2022 Aug 23;12:879876. doi: 10.3389/fonc.2022.879876 (PMC9448664; doi:10.3389/fonc.2022.879876)
Supplement: Supplementary file 1 [file Table_1.pdf]

**Supplement:**

**Table S. 1 Characteristics of patients with and without seroconversion**

| | **Seroconversion N (%)** | **No seroconversion N (%)** |
|---|---|---|
| **Total** | **35 (100.0)** | **6 (100.0)** |
| Median age, years (range) | 64.0 (41-78) | 73.5 (45-80) |
| Sex | | |
| Female | 16 (45.7) | 1 (16.7) |
| Male | 19 (54.3) | 5 (83.3) |
| Type of skin cancer | | |
| Melanoma | 32 (91.4) | 5 (83.3) |
| Merkel cell carcinoma | 2 /5.7) | 1 (16.7) |
| Squamous cell carcinoma | 0 (0.0) | 0 (0.0) |
| Basal cell carcinoma | 1 (2.9) | 0 (0.0) |
| Charlson comorbidity index[1] | | |
| 0 | 30 (85.7) | 1 (16.7) |
| 1-2 | 5 (14.3) | 2 (33.3) |
| ≥3 | 0 (0.0) | 1 (16.7) |
| Overall performance status (ECOG) | | |
| 0 | 34 (97.1) | 1 (16.7) |
| 1-2 | 1 (2.9) | 4 (66.7) |
| ≥3 | 0 (0.0) | 1 (16.7) |
| LDH (serum) | | |
| Normal | 31 (88.6) | 4 (66.7) |
| Increased | 4 (11.4) | 2 (33.3) |
| Number of organs involved | | |
| 0 | 13 (37.1) | 1 (16.7) |
| 1-3 | 19 (54.3) | 4 (66.7) |
| >3 | 3 (8.6) | 1 (16.7) |
| Type of systemic treatment | | |
| Immune checkpoint inhibition | 29 (82.9) | 6 (100.0) |
| Monotherapy (PD-1, PD-L1) | 22 (62.9) | 4 (66.7) |
| Combination (CTLA-4+PD-1) | 7 (20.0) | 2 (33.3) |
| Targeted therapy (BRAF+MEK) | 5 (14.3) | 0 (0.0) |
| Chemotherapy | 1 (2.9) | 0 (0.0) |
| Median number of immunotherapy cycles (range) | 9 (1-26) | 8 (1-23) |
| Treatment setting | | |
| Adjuvant | 13 (37.1) | 1 (16.7) |
| Non-adjuvant | 22 (62.9) | 5 (83.3) |
| Treatment line | | |
| First-line | 23 (65.7) | 4 (66.7) |
| Second-line or higher | 12 (34.3) | 2 (33.3) |
| Tumor response to systemic treatment | | |
| Complete response | 2 (5.7) | 0 (0.0) |
| Partial response | 10 (28.6) | 1 (16.7) |
| Stable disease | 3 (8.6) | 1 (16.7) |
| Progressive disease | 5 (14.3) | 4 (66.7) |
| No evidence of disease | 12 (34.3) | 0 (0.0) |
| Not evaluable | 3 (8.6) | 0 (0.0) |
| Survival status | | |
| Alive | 35 (100.0) | 5 (83.3) |
| Dead | 0 (0.0) | 1 (16.7) |
| Died from skin cancer | 0 (0.0) | 1 (16.7) |
| Died from COVID-19 | 0 (0.0) | 0 (0.0) |

1, modified, the underlying skin cancer was excluded from comorbidities.
